# MRI-based brain tumor detection using convolutional deep learning methods and chosen machine learning techniques

**DOI:** 10.1186/s12911-023-02114-6

**Published:** 2023-01-23

**Authors:** Soheila Saeedi, Sorayya Rezayi, Hamidreza Keshavarz, Sharareh R. Niakan Kalhori

**Affiliations:** 1 Medical Informatics and Health Information Management Department, School of Allied Medical Sciences, Tehran University of Medical Sciences, 3rd Floor, No #17, Farredanesh Alley, Ghods St, Enghelab Ave, Tehran, 14177-44361 Iran; 2 Faculty of Electrical and Computer Engineering, Tarbiat Modares University, Tehran, Iran; 3 Peter L. Reichertz Institute for Medical Informatics, TU Braunschweig and Hannover Medical School, 38106 Brunswick, Germany

**Keywords:** Convolutional neural network, Brain tumor, Machine learning, Medical imaging

## Abstract

**Background:**

Detecting brain tumors in their early stages is crucial. Brain tumors are classified by biopsy, which can only be performed through definitive brain surgery. Computational intelligence-oriented techniques can help physicians identify and classify brain tumors. Herein, we proposed two deep learning methods and several machine learning approaches for diagnosing three types of tumor, i.e., glioma, meningioma, and pituitary gland tumors, as well as healthy brains without tumors, using magnetic resonance brain images to enable physicians to detect with high accuracy tumors in early stages.

**Materials and Methods:**

A dataset containing 3264 Magnetic Resonance Imaging (MRI) brain images comprising images of glioma, meningioma, pituitary gland tumors, and healthy brains were used in this study. First, preprocessing and augmentation algorithms were applied to MRI brain images. Next, we developed a new 2D Convolutional Neural Network (CNN) and a convolutional auto-encoder network, both of which were already trained by our assigned hyperparameters. Then 2D CNN includes several convolution layers; all layers in this hierarchical network have a 2*2 kernel function. This network consists of eight convolutional and four pooling layers, and after all convolution layers, batch-normalization layers were applied. The modified auto-encoder network includes a convolutional auto-encoder network and a convolutional network for classification that uses the last output encoder layer of the first part. Furthermore, six machine-learning techniques that were applied to classify brain tumors were also compared in this study.

**Results:**

The training accuracy of the proposed 2D CNN and that of the proposed auto-encoder network were found to be 96.47% and 95.63%, respectively. The average recall values for the 2D CNN and auto-encoder networks were 95% and 94%, respectively. The areas under the ROC curve for both networks were 0.99 or 1. Among applied machine learning methods, Multilayer Perceptron (MLP) (28%) and K-Nearest Neighbors (KNN) (86%) achieved the lowest and highest accuracy rates, respectively. Statistical tests showed a significant difference between the means of the two methods developed in this study and several machine learning methods (*p*-value < 0.05).

**Conclusion:**

The present study shows that the proposed 2D CNN has optimal accuracy in classifying brain tumors. Comparing the performance of various CNNs and machine learning methods in diagnosing three types of brain tumors revealed that the 2D CNN achieved exemplary performance and optimal execution time without latency. This proposed network is less complex than the auto-encoder network and can be employed by radiologists and physicians in clinical systems for brain tumor detection.

## Introduction

In medical terms, tumors are known as malignant or benign neoplasms, of which there are more than 200 diverse varieties that may affect humans [1]. According to the American Cancer Society, a brain tumor is a severe disease in which irregular brain tissue growth impairs brain function. The National Brain Tumor Foundation (NBTF) reported that the number of people who have lost their lives due to brain tumors has increased by 300% in the last three decades [2]. Brain tumors can lead to death if left untreated [3]. The complexity of brain tumors poses challenges for healthcare providers in diagnosing and caring for affected patients. Early detection of brain tumors and initiation of treatment play vital roles in the survival rate of these patients [4]. Brain tumor biopsy is not as easy as biopsy of other parts of the body, as it must be performed with surgery. Therefore, the need for another method for accurate diagnosis without surgery is crucial. Magnetic Resonance Imaging (MRI) is the best and most commonly used option for diagnosing brain tumors [5].

Recent advances in machine learning, particularly in deep learning, have led to the identification and classification of medical imaging patterns. Successes in this area include the possibility of retrieving and extracting knowledge from data instead of learning from experts or scientific texts. Machine learning is rapidly becoming a helpful tool for improving performance in various medical applications in various fields, including the prognosis and diagnosis of diseases, identification of molecular and cellular structures, tissue segmentation, and the classification of images [6–8]. In image processing, the most successful techniques currently used are Convolutional Neural Networks (CNNs), as they have many layers and high diagnostic accuracy if the number of input images is high [9, 10]. Autoencoders are an unsupervised learning method in which neural networks are leveraged for representation learning. Remarkably, various deep learning and machine learning algorithms have been used to identify tumors (such as lung tumors) and detect cardiovascular stenosis. Moreover, performance evaluations have shown that they have high diagnostic accuracy [11–14].

Many studies have been conducted on the detection of brain tumors by various methods and models [5, 15–21]. However, some of these studies have had a number of limitations, such as a lack of a performance comparison between the proposed model and traditional machine learning methods [5, 22, 23]. The proposed model in one study required complex computations [24]. The majority of relevant studies have provided models for classifying three types of brain tumors without including healthy subjects [22–25].

Speaking scientifically, tumor diagnosis by medical images is erroneous and depends heavily on the radiologist's experience. Because of widespread pathology variation and the possible fatigue of human specialists, researchers and physicians can benefit from computer-assisted interventions [6], and computational intelligence-oriented techniques can assist physicians in identifying and classifying brain tumors [5]. Machine learning approaches, especially deep learning, can also play a vital role in the analysis, segmentation, and classification of cancer images, especially brain tumors [26]. Furthermore, the use of such methods paves the way for accurate and error-free identification of tumors to recognize and distinguish them from other similar diseases. In the present study, we have tried to propose models that consider the suggestions and limitations presented in studies and suggest suitable solutions for them. Eight modeling methods have been compared to determine whether a significant difference exists between these methods in terms of performance.

### Contributions of this work

The significant contributions of this work are detailed below:

1. Our networks are performed on an extensive dataset of 3264 T1-weighted contrast-enhanced MRI images, which are desirable for the training and testing phases.
2. The internal architecture of the modified 2D CNN and convolutional auto-encoder neural network are adjusted in terms of the number of layers, how the layers are positioned next to each other, the type of parameters and hyperparameters, and their values that can be varied to fine-tune our models to enhance accuracy.
3. Extracted essential features are utilized to classify three types of brain tumors and healthy brains (no tumor) by 2D CNN, auto-encoder network, and six common machine learning techniques.
4. In the modified 2D CNN, several convolution layers are considered; all layers in this hierarchical network have a 2*2 kernel function. This network consists of eight convolutional layers and four pooling layers; after all convolution layers, batch-normalization layers were applied. The training process was accomplished over 100 training epochs, and the batch size was 16. Each epoch last 7 s.
5. The auto-encoder network includes a convolutional auto-encoder network and a convolutional network for classification that uses the last output encoder layer of the first part. The encoder part has a convolutional layer of 32-filter length, two continuous convolutional layers with a filter length of 128, and two continuous convolutional layers with a filter length of 64. The decoding part of the network consists of a convolutional layer with a filter length of 32, two continuous convolutional layers with a filter length of 64, and two continuous convolutional layers with a filter length of 128 as well as a convolutional layer with a filter length of 128. For all convolution layers, the 2*2 kernel function was applied.
6. The developed networks achieved optimal accuracy of approximately 95% to 96%, and areas under the receiver operating characteristics curves (AUROC) are 0.99 or 1. Performance analysis proposes a renovation of our proposed techniques by comparing related papers.
7. One-way ANOVA for three parameters of precision, recall, and F-measure in eight modeling methods showed a statistically significant difference between the methods (*p*-value < 0.001).
8. Our architectures attain competitive undertakings analogized with other state-of-the-art approaches on the MRI dataset and demonstrate a heightened generalization.

### Related works

In recent years, many methodologies for classifying brain tumors by MRI images have been developed (Table 1).

**Table 1:** Studies conducted on brain tumor detection

References	Purpose	Model	Limitations/future works
Badža et al. [5]	To classify different types of brain tumors using a convolutional neural network	CNN	Examining the execution of the designed neural network in the mentioned study, as well as enhanced ones in different medical images
Gumaei et al. [23]	To classify brain tumors using a hybrid feature extraction method	RELM	Lack of comparison of the technique used in this study and other machine learning methods
Rehman et al. [22]	Proposing three architectures of convolutional neural networks (alexnet,Googlenet, and vggnet) to classify brain tumors	Convolutional neural networks (AlexNet, GoogLeNet, and VGGNet)	Explore other essential deep neural network’s architectures for brain tumor classification with less time complexity
Mittal et al. [29]	Using segmentation method to diagnose brain tumors using deep learning-based methods	Combination of SWT and GCNN	Other databases like PASCAL, Berkeley or BRATS can be used
			It is recommended to use a variety of diverse classifiers to increase the accuracy of the classifier
Phaye et al. [24]	Provide an approach to improve outputs using a network with dense layers	Dense capsule networks (DCNet) and diverse capsule networks (DCNet++)	Computational complexity must be reduced to enhance classifier execution
Pashaei et al. [27]	Developing an algorithm for extracting and classifying features with the CNN and KELM	KELM	Not mentioned
Abiwinanda et al. [28]	Use the convolutional neural network to segment and classify brain tumors automatically	CNN	Pay attention to the color balancing step to improve the classifier's accuracy
Abd-Ellah et al. [15]	Brain tumor detection with a two-step automatic detection system	Preprocessing, feature extraction using CNN and classification with error-correcting output codes support vector machine (ECOC-SVM)	Not mentioned

A study conducted by Badža and Barjaktarovic´ in 2020 used a CNN to classify glioma, meningioma, and pituitary tumors. The network architecture applied in this study consisted of an input layer, two blocks “A,” two blocks “B,” a classification block, and an output layer, with 22 layers in total. Network performance was evaluated by employing the k-fold cross-validation method. The best value for the tenfold cross-validation method, which was obtained in this study, was 96.56%. The image dataset used in this study comprised 3064 T1-weighted contrast-enhanced MRI images from the Nanfang Hospital, General Hospital, and Tianjin Medical University in China [5].

In 2018 [24] developed capsule algorithms networks (DCNet) and diverse capsule networks (DCNet++). DCNet essentially adds a deeper convolutional network, leading to learning distinctive feature maps. DCNet++ uses a hierarchical architecture for learning, which makes it more efficient for learning complex data. They used a dataset comprising 3064 MRI images of 233 brain tumor patients for classification and considered only images of three types of brain tumors; a dataset of healthy people was not considered for classification. The DCNet model was developed by changing the eight initial convolutional layers to four layers with 16 kernels and was trained with eightfold cross-validation. The accuracy of the DCNet algorithm test was 93.04%, and the accuracy of the DCNet++ algorithm was 95.03%.

Gumaei et al. [23] introduced an automated approach to assist radiologists and physicians in identifying different types of brain tumors. The study was conducted in three steps: brain image preprocessing, brain feature extraction, and brain tumor classification. In the preprocessing step, brain images were converted into intensity brain images in the range of [0, 1], using a min–max normalization rule. In the next step, the PCA-NGIST method (a combination of normalized GIST descriptor with PCA) was adopted to extract features from MRI images. In the final step, Regularized Extreme Learning Machine (RELM) classification was applied to identify and classify the tumor types. The dataset provided by Cheng was used by the researchers in their study and consisted of 3064 MRI images from 233 patients divided into two subsets, 70% was used for training and 30% for classifier testing; a fivefold cross-validation method was utilized. The results reported 94.23% accuracy. The study, however, performed no comparative evaluation with other techniques, which can be considered as a study limitation [23].

Pashaei et al. [27] developed different methods to identify meningioma, glioma, and pituitary tumors. In their model, a CNN was used to extract hidden features from images and select features. The proposed model consisted of four convolutional layers, four pooling layers, one fully connected layer, and four batch normalization layers. The authors used ten epochs, 16 iterations per epoch, and the learning rate in this model was 0.01. The dataset provided by Cheng was also used in this study. The performance of the proposed model was evaluated using a tenfold cross-validation method, and 70% and 30% of the data was applied for training and system testing, respectively. The study compared the proposed method with MLP, Stacking, XGBoost, SVM, and RBF, and the results showed the high accuracy of the proposed method (93.68%) [27].

A CNN was also used by Abiwinanda in 2018 to diagnose the three most common types of brain tumors. In the learning process, the “adam” optimizer was used, which is a method for stochastic optimization using the stochastic gradient descent principle. In the study, the CNN was trained by 3064 T-1 weighted CE-MRI from brain tumor images provided by Cheng. The dataset included 1426 images of meningiomas, 708 images of gliomas, and 930 images of pituitary tumors. Of all the available images, 700 images from each class were applied, of which 500 were used for the training phase, and another 200 images were considered for the validation phase. In this model, all convolutional layers in the architectures used 32 filters, ReLu was used as an activation function, the maxpool kernel size was 2 × 2, and all the fully connected layers used 64 neurons. There were three neurons in the output layer, and the softmax activation function was employed at the output layer. The best-reported accuracy rates for training and validation were 98.51% and 84.19%, respectively [28].

In another study (2018), CNNs were applied to diagnose brain tumors using magnetic resonance images automatically. This study aimed to differentiate between healthy brains and brain tumor images. A two-stage multi-model system made the diagnosis. In the first stage, preprocessing and feature selection were performed by a CNN, and in the second stage, classification was done by an Error-Correcting Output Codes Support Vector Machine (ECOC-SVM). In the first stage, three algorithms, namely AlexNet, VGG-16, and VGG-19, were employed, among which AlexNet had the best performance with 99.55% accuracy. BraTS (2013 dataset) was used for the brain tumor localization phase, and images extracted from the standard Reference Image Database to Evaluate Response (RIDER) neuro MRI database were used for performance evaluation in the first phase [15].

Rehman et al. [22] studied three CNNs, namely AlexNet, GoogLeNet, and VGGNet. The study's primary purpose was to differentiate three brain tumor types, meningioma, glioma, and pituitary, using deep learning techniques and MRI images processing. Automated features were classified in the last phase using a linear classifier. Data augmentation techniques were applied to increase the sample size and reduce the possibility of over-fitting. The evaluation results showed that the VGG16 technique had the highest accuracy (98.69%) compared to other methods [22].

Mittal et al. [29] used the combination of Stationary Wavelet Transform (SWT) and a new Growing CNN (GCNN) to automate the segmentation process. In fact, they utilized these effective methods to identify brain tumors by MRI images. The evaluation results showed that the technique proposed in the study had the highest accuracy compared to the genetic algorithm; K-NN, SVM, and CNN [29, 30].

Paul et al. [25] used deep learning methods to classify brain images related to meningioma, glioma, and pituitary tumors. In this research, the same dataset, i.e., 3064 T1-weighted contrast-enhanced MRI brain images of 233 patients, was applied; two types of neural networks, i.e., fully connected and CNNs, were designed. Moreover, a fivefold cross-validation technique showed that the general methods, with an accuracy of 91.43%, worked better than the specific methods, which required image dilation [25].

## Material and methods

**Fig. 1:**
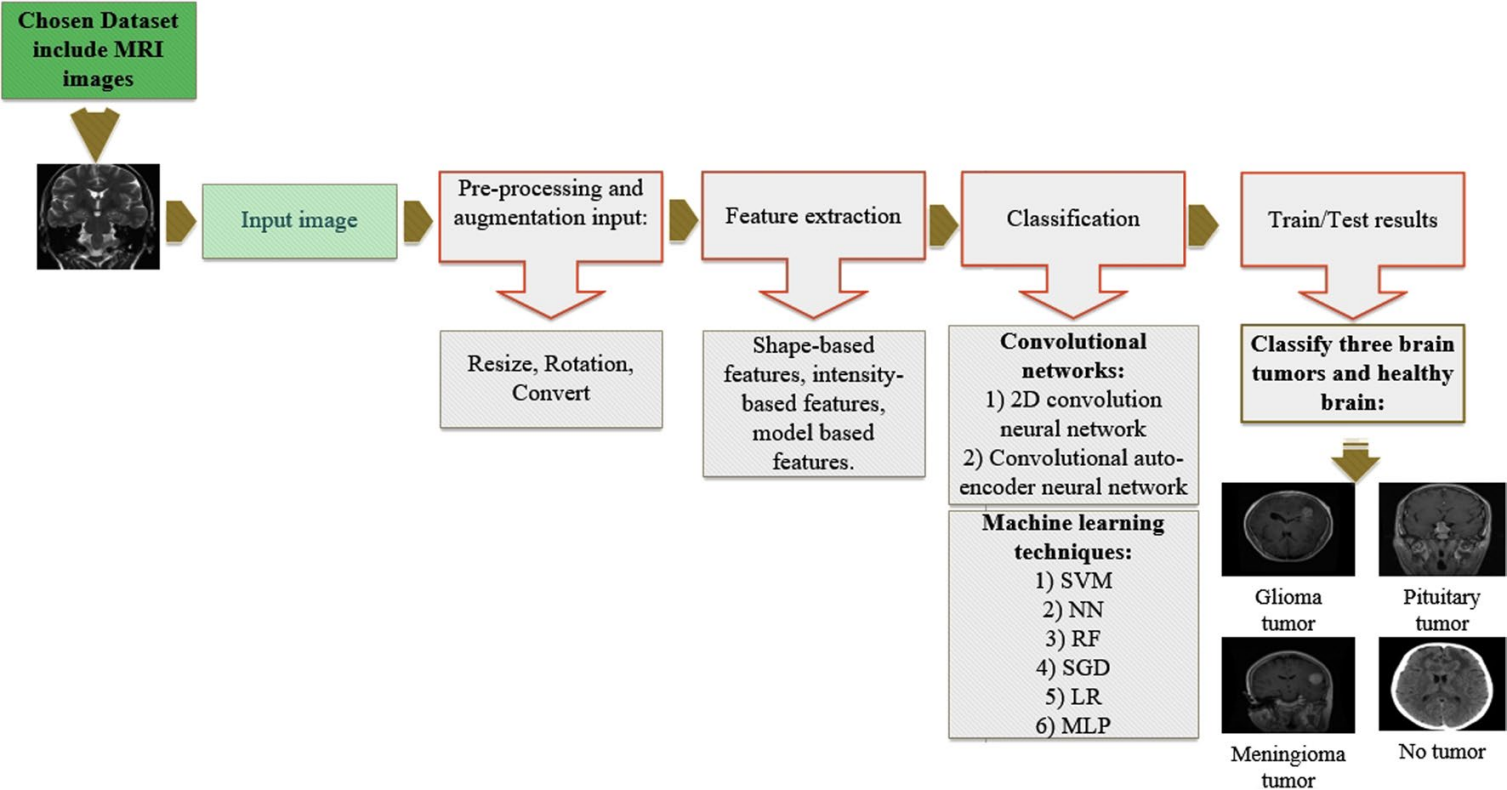
Stages of the proposed methodology

The methodology of the present study is illustrated in Fig. 1. Major steps in the present study comprise brain tumor dataset selection, pre-processing MRI images, feature extraction, and classification by various classifiers.

### Dataset

**Fig. 2:**
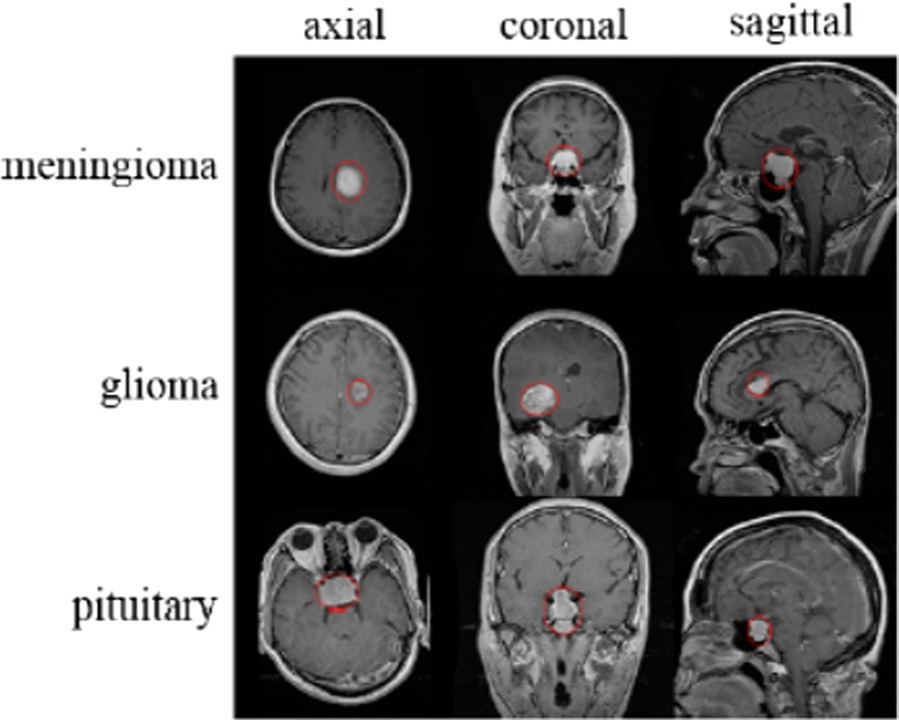
Description of normalized MRI images presenting diverse varieties of tumor in a different plane

The applied image-based dataset comprised 3264 T1-weighted contrast-enhanced MRI images [31]. There were four types of images in this dataset: glioma (926 images), meningioma (937 images), pituitary gland tumor (901 images), and healthy brain (500 images). All images were in sagittal, axial, and coronal planes. Figure 2 presents examples of the various types of tumors and different planes. The segment of tumors has been branded with a red outline. The number of images is different for each patient.

### Data augmentation and image pre-processing

Magnetic resonance images from this dataset had distinct sizes. These images represented the networks' input layer, so they were resized to 80*80 pixels. Each image was converted in two directions to augment the dataset. The first change included image rotation by 90°, and the second was flipping images vertically. Our chosen dataset was augmented three times, which resulted in 9792 images.

### Proposed solutions

#### 2D CNN

**Fig. 3:**
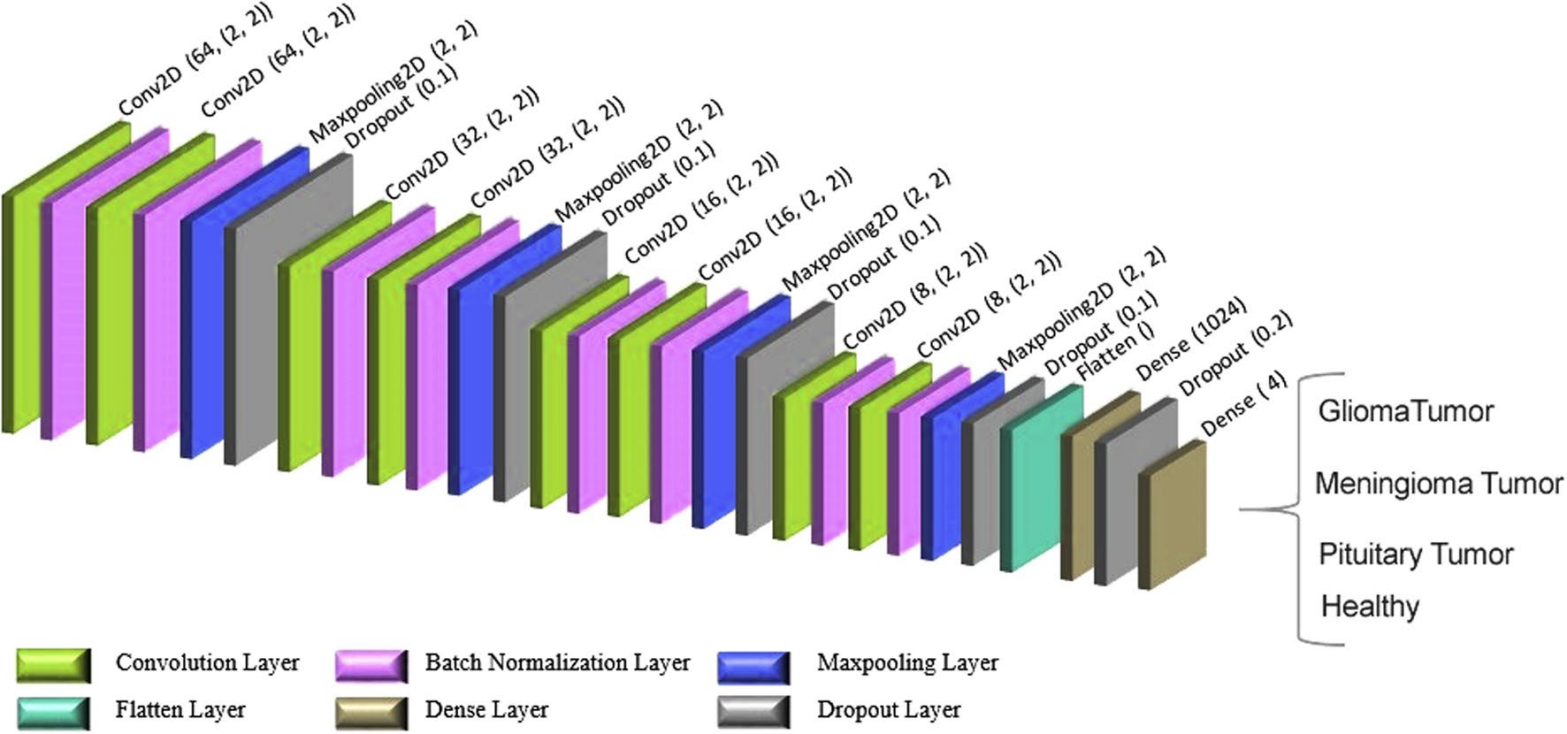
The architecture of the 2D convolution network

Figure 3 shows the proposed architecture for the two-dimensional CNN. A set of 9792 data was used in this study, 90% (8812) of which was employed as the training data and 10% (980) as the testing data. The proposed network had several layers, including convolution, which possessed two convolutional layers with 64 filters. Moreover, two convolution layers included 32 filters, and the others have 16. The final two convolutional layers make the desired network filters with a length of 8. The layers in this network have a 2*2 kernel function.

The convolutional network, which is also referred to as a neural network, has a hierarchical structure. This network creates a link between convolution layers, alternate pooling layers, and fully connected layers. One factor that should be noted here is that there is no need to use a pooling layer after each convolution layer. Figure 3 shows that the network has eight convolutional and four pooling layers. The final pooling layer with 2D output is changed to a 1D layer by flattened layers so it can be sent to the fully connected layers. Also, a type of padding is needed to manage and control the convolutional layer's output size. This study showed that the padding in adjacent cells is used for all networks to manage the edges of input data with the same values. To classify the data into categories by softmax activation function, a total of 1024 fully connected layer and a 4 fully connected layer were used. In this process, the batch-normalization layers were used to prevent overfitting. A dropout layer with a rate of 0.1 was also used following the max-pooling and fully connected layers.

For the activation function, the ReLU function was used in all layers apart from the last fully connected layer. To increase the efficiency, the Adam was used as an optimizing function. Different values, including 0.01, 0.001 and 0.0001, were used to test the learning rate parameter. Also, the best value with minimum learning error was found to be 0.001.

After 100 epochs, the training process was confirmed. The batch size was determined to be 16, and each epoch lasted about 7 s. The features extracted from the convolutional layer included input from the first layer fully connected to Ufc = 1024 hidden layers. The number of weights (Wconv) depended on the output size of the prior convolution layer (y1*y2), the number of filters (k), and the number of hidden layers in fully connected layers. Thus, the convolutional layer's weight was determined as follows [32]: Wconv = y1*y2*k*Ufc = 5*5*8*1024 = 204,800, where the number of existing parameters to the first fully connected layer equals 204,800 + 1024 (biases) = 205,824.

A summary of learning parameters for the proposed network can be seen in Table 2. As seen in this table, the value of all parameters used to determine the four categories of this network are calculated by summing up the values in cited in the param column in Table 2. The consequent value is 243,924, where all parameters are trainable.

**Table 2:** Modified parameters in the convolution network to classify the 4 categories

Model: “sequential”		
Layer (type)	Output shape	Param #
Conv2d (Conv2D)	(None, 80, 80, 64)	832
Batch_normalization (BatchNormalization)	(None, 80, 80, 64)	256
Conv2d_1 (Conv2D)	(None, 80, 80, 64)	16,448
Batch_normalization_1 (BatchNormalization)	(None, 80, 80, 64)	256
Max_pooling2d (MaxPooling2D)	(None, 40, 40, 64)	0
dropout (Dropout)	(None, 40, 40, 64)	0
Conv2d_2 (Conv2D)	(None, 40, 40, 32)	8224
Batch_normalization_2 (BatchNormalization)	(None, 40, 40, 32)	128
Conv2d_3 (Conv2D)	(None, 40, 40, 32)	4128
Batch_normalization_3 (BatchNormalization)	(None, 40, 40, 32)	128
Max_pooling2d_1 (MaxPooling2D)	(None, 20, 20, 32)	0
Dropout_1 (Dropout)	(None, 20, 20, 32)	0
Conv2d_4 (Conv2D)	(None, 20, 20, 16)	2064
Batch_normalization_4 (BatchNormalization)	(None, 20, 20, 16)	64
Conv2d_5 (Conv2D)	(None, 20, 20, 16)	1040
Batch_normalization_5 (BatchNormalization)	(None, 20, 20, 16)	64
Max_pooling2d_2 (MaxPooling2D)	(None, 10, 10, 16)	0
Dropout_2 (Dropout)	(None, 10, 10, 16)	0
Conv2d_6 (Conv2D)	(None, 10, 10, 8)	520
Batch_normalization_6 (BatchNormalization)	(None, 10, 10, 8)	32
Conv2d_7 (Conv2D)	(None, 10, 10, 8)	264
Batch_normalization_7 (BatchNormalization)	(None, 10, 10, 8)	32
Max_pooling2d_3 (MaxPooling2D)	(None, 5, 5, 8)	0
Dropout_3 (Dropout)	(None, 5, 5, 8)	0
Flatten (Flatten)	(None, 200)	0
Dense (Dense)	(None, 1024)	205,824
Dropout_4 (Dropout)	(None, 1024)	0
Dense_1 (Dense)	(None, 4)	4100

#### Convolutional auto-encoder neural network

**Fig. 4:**
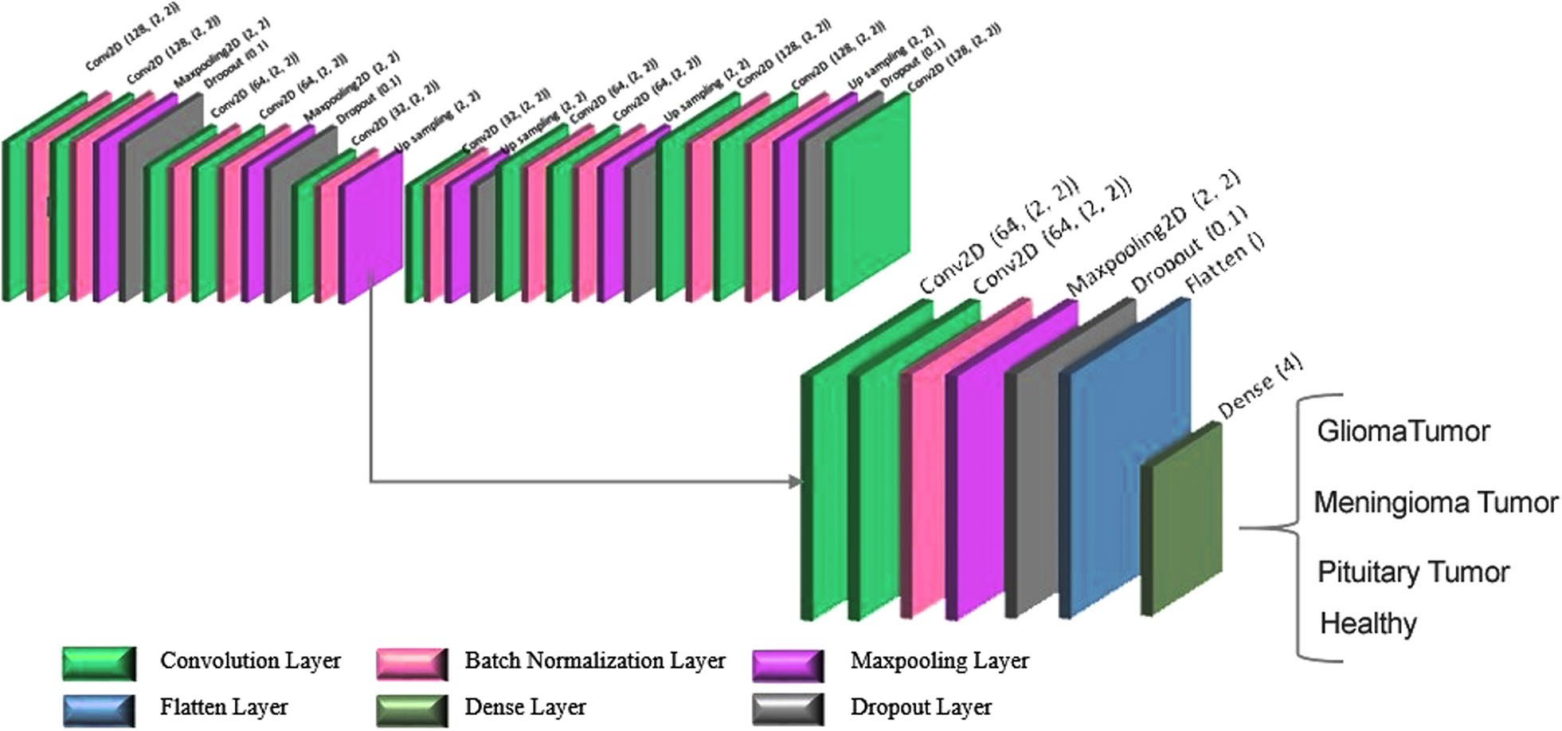
Convolutional auto-encoder network classification part

This study was conducted to design the architecture of a convolutional auto-encoder network. In this network, in order to predict the target value (Y) for the input (X), an auto-encoder was trained to predict the input (X) rather than training the network. The auto-encoder network was used to train and classify the data set instead of creating input images. Figure 4 shows the designed architecture of the convolutional auto-encoder network.

The network architecture designed in this study had two main parts. The first part included the convolutional auto-encoder network for data training, and the second part contained a convolutional network for classification, which utilizes the last output encoder layer of the first part. The first part of the architecture also consisted of 2D multilayer convolutional networks for both the encoder and the decoder. A total data set of 9792 was used in this study, 90% (8812) of which was used as the training data and 10% (980) as the test data.

The encoder part included a convolutional layer with a 32-filter length, two continuous convolutional layers with a 128-filter length, and two continuous convolutional layers with a 64-filter length. In the encoder, no pooling layer existed after each convolutional layer, but a second stage 2*2 max-pooling layer was considered after a sequence of two convolutional layers. The network’s decoder also included a convolutional layer with a 32-filter length, two continuous convolutional layers with a 64-filter length, two continuous convolutional layers with a 128-filter length, and a convolutional layer with a 128-filter length. A 2*2 kernel function was used for all convolutional layers, and there was no up-sampling layer after each convolutional layer. However, after a sequence of two convolutional layers, a 2*2 up-sampling layer was applied. The same padding was used for this network, and a batch normalization layer after each convolutional layer was considered. A dropout of 0.1 was also operated after each max-pooling layer, apart from the last layer, to prevent overfitting. Values of 0.01, 0.001 and 0.0001 were used to examine the learning rates, and the best value with minimum learning error was found to be 0. 001. The designed network was trained after 100 training epochs, and data was transmitted to the network in batches of 16 (batch-size), while each epoch ran in 14 s.

**Fig. 5:**
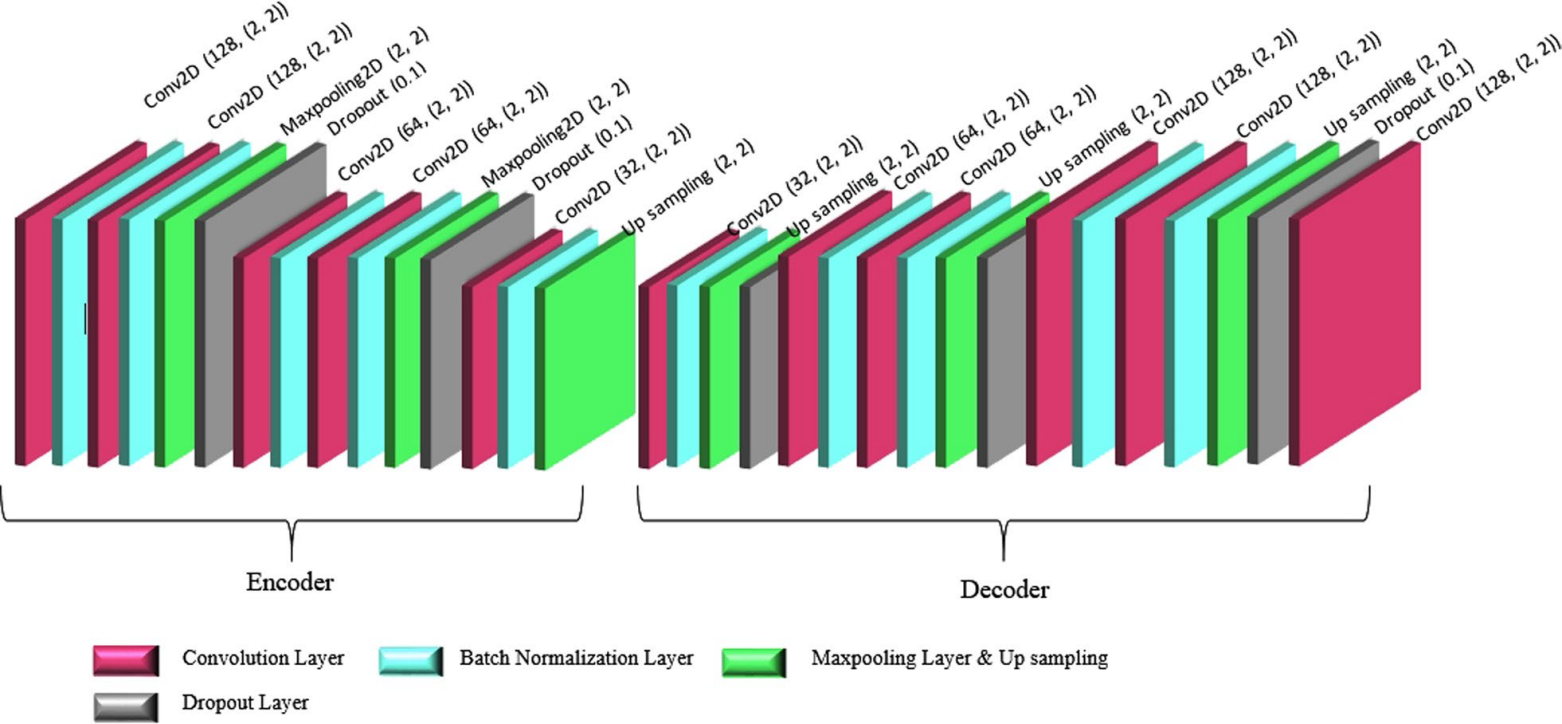
Architecture of the proposed convolutional auto-encoder network

The critical features of the input data were removed by the automatic encoder network, and the output of the encoder layer was used for the classification (Fig. 5). For accurate classification, the output of the encoder layer was trained by two continuous convolution layers with 64-filter length, a 2*2-kernel function, and a 2*2-max-pooling layer with step 2. Batch-normalization and 0.1 dropout layers were also used to prevent overfitting [33]. To forward the output of the max-pooling layer to a 4-fully connected layer, the flattened layer was used [34], and the ReLU activation function was used for all layers.

The vital factors for training and classification in the auto-encoder convolutional network include the encoder and classifier parameters. The extracted features of the encoder's last layer are trained by several convolutional layers, and the final extracted features would turn into the input of the first layer fully connected to the hidden layer of Ufc = 4. The number of weights (Wconv) depends on the number of hidden layers in the fully connected layer and the output size of the flattened layer. The flattened layer's output equals 5*5*64 = 1,600. Hence, the number of weights equals Wconv = out-flatten*Ufc = 1600*4 = 6,400, and the number of existing parameters to the second fully connected layer equals 6400 + 4 (biases) = 6404.

The learning parameters of this network are presented in Table 3. The value of all modified parameters can be calculated by summing up the values in the param column (Table 3). The value of all modified parameters is 158,760, of which 1,569,000 are related to learning, and 960 are related to non-learning parameters.

**Table 3:** Modified parameters in the convolutional auto-encoder network for the classification of four categories

Layer (type)	Output shape	Param #
Input_1 (InputLayer)	(None, 80, 80, 3)	0
Conv2d (Conv2D)	(None, 80, 80, 128)	1664
Batch_normalization (BatchNormalization)	(None, 80, 80, 128)	512
Conv2d_1 (Conv2D)	(None, 80, 80, 128)	65,664
Batch_normalization_1 (BatchNormalization)	(None, 80, 80, 128)	512
Max_pooling2d (MaxPooling2D)	(None, 40, 40, 128)	0
Dropout (Dropout)	(None, 40, 40, 128)	0
Conv2d_2 (Conv2D)	(None, 40, 40, 64)	32,832
Batch_normalization_2 (BatchNormalization)	(None, 40, 40, 64)	256
Conv2d_3 (Conv2D)	(None, 40, 40, 64)	16,448
Batch_normalization_3 (BatchNormalization)	(None, 40, 40, 64)	256
Max_pooling2d_1 (MaxPooling2D)	(None, 20, 20, 64)	0
Dropout_1 (Dropout)	(None, 20, 20, 64)	0
Conv2d_4 (Conv2D)	(None, 20, 20, 32)	8224
Batch_normalization_4 (BatchNormalization)	(None, 20, 20, 32)	128
Max_pooling2d_2 (MaxPooling2D)	(None, 10, 10, 32)	0
Conv8 (Conv2D)	(None, 10, 10, 64)	8256
Conv2d_11 (Conv2D)	(None, 10, 10, 64)	16,448
Batch_normalization_10 (BatchNormalization)	(None, 10, 10, 64)	256
Max_pooling2d_7 (MaxPooling2D)	(None, 5, 5, 64)	0
Dropout_5 (Dropout)	(None, 5, 5, 64)	0
Flatten (Flatten)	(None, 1600)	0
Dense (Dense)	(None, 4)	6404
Total parameters: 157, 860		
Trainable parameters: 156, 900		
Non-trainable parameters: 960		

Whole process in the present study was carried out in Keras with the Tensorflow backend. The networks in this study were designed in the Python environment and then, ran by cross-library in the Google Collaboratory (Colab) environment. Colab supplies a platform for running Python codes, especially machine learning, deep learning, and data analysis. The details of Colab hardware technical characteristics are given in Table 4.

**Table 4:** Colab hardware specifications

Hardware	Description
GPU	1 × Tesla K80, compute 3.7, having 2496 CUDA cores, 12 GB GDDR5 VRAM
CPU	1 × single core hyper threaded Xeon Processors @2.3Ghz i.e. (1 core, 2 threads)
RAM	~ 12.6 GB available
Disk	~ 33 GB available

### Performance evaluation metrics

The main objective of the current study was to classify MRI images into glioma, meningioma, pituitary gland tumor, and healthy brain classes. Metrics for performance evaluation included accuracy, precision, recall, and F-measure.

Accuracy refers to the proximity of a measured value to a standard or actual value. In other words, it is the ability of the tool to measure the exact value, whose accuracy can be measured.

1 $${\text{Accuracy}} = \frac{{\textit{TP}} + {\textit{TN}}}{{{\textit{TP}} + {\textit{TN}} + {\textit{FP}} + {\textit{FN}}}}$$

In machine learning, precision results from dividing actual cases into sums of true and false cases. Recall is also the result of dividing the true items by all the items in that class. The weighting value for F-measure can be computed based on the precision and recall measures. F-measure is a good measure in evaluating the quality of classification and describing the weighted average between the quantities of precision and recall. The value of this measure is between 0 and 1, with 0 being the worst circumstance and 1 the best condition. This parameter was calculated by the following Eq. (4):

2 $${\text{Precision}} = \frac{{\textit{TP}}}{{{\textit{TP}} + {\textit{FP}} }}$$

3 $${\text{Recall or Sensitivity}} = \frac{{\textit{TP}}}{{{\textit{TP}} + {\textit{FN}} }}$$

4 $${\text{F - measure}} = 2 \times \frac{{\textit{Precision}} \times {\textit{Recall}}}{{{\textit{Precision}} + {\textit{Recall}}}}$$

For organizing and evaluating classifiers and visualizing their performance, drawing receiver operating characteristics (ROC) plots can be useful in describing the results. ROC plots are commonly applied in medical decision-making and have recently been noticed in machine learning and data mining. The ROC curve is constructed by plotting the true positive rate (TPR) versus the false positive rate (FPR) in various threshold sets. Therefore, maximizing TPR while minimizing FPR are ideal achievements. This means that the upper left corner of the plot is the ideal point (FPR = 0 and TPR = 1).

## Experimental results

**Fig. 6:**
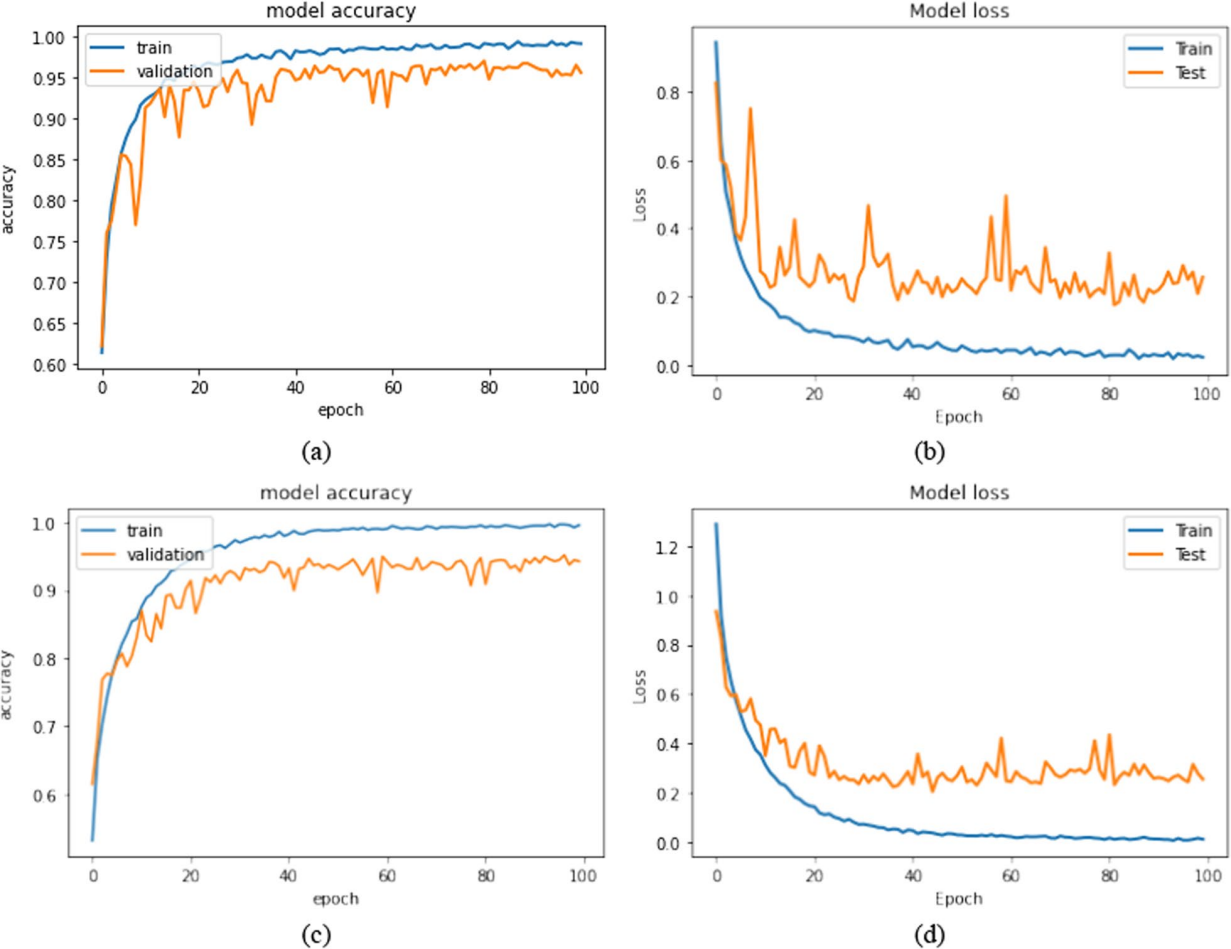
Training and validation analysis over 100 epochs for (1) 2D CNN: **a** training and testing accuracy analysis, and **b** training and testing loss analysis. (2) Convolutional auto-encoder neural network: **c** training and testing accuracy analysis, and **d** training and testing loss analysis

Table 5 outlines the results of our proposed 2D CNN and convolutional auto-encoder neural network. The training accuracy of the proposed 2D CNN was found to be 96.4752%, whereas its validation accuracy was 93.4489%. The training accuracy of the proposed convolutional auto-encoder was found to be 95.6371%, and its validation accuracy was 90.9255%. The precision, recall, and F-measure of the four classes obtained from 2D CNN and the convolutional auto-encoder neural network are summarized in Tables 6 and 7, respectively. Figure 6 shows the training, validation accuracy, and loss analyses of the proposed models concerning the number of epochs.

**Table 5:** Network results for classification into 4 classes

Network	Training-accuracy	Test-accuracy	Train-loss	Test-loss
2D CNN	0.96475260	0.93448979	0.093299804	0.28095046
Convolutional auto-encoder	0.95637199	0.90925510	0.11472394	0.32122133

**Table 6:** Precision, recall, and F-measure of 2D CNN

	Precision	Recall	F-measure
Glioma	0.95	0.95	0.95
Meningioma	0.96	0.93	0.94
Pituitary gland tumor	**0.97**	0.97	**0.97**
Healthy brain	0.91	**0.98**	0.94
Average	0.9475	0.9575	0.95

We bolded the parameters that had the best performance for each group

**Table 7:** Precision, recall, and F-measure of convolutional auto-encoder neural network

	Precision	Recall	F-measure
Glioma	0.95	0.94	0.94
Meningioma	0.93	0.94	0.93
Pituitary gland tumor	**0.96**	**0.97**	**0.97**
Healthy brain	0.93	0.92	0.92
Average	0.9425	0.9425	0.94

We bolded the parameters that had the best performance for each group

**Fig. 7:**
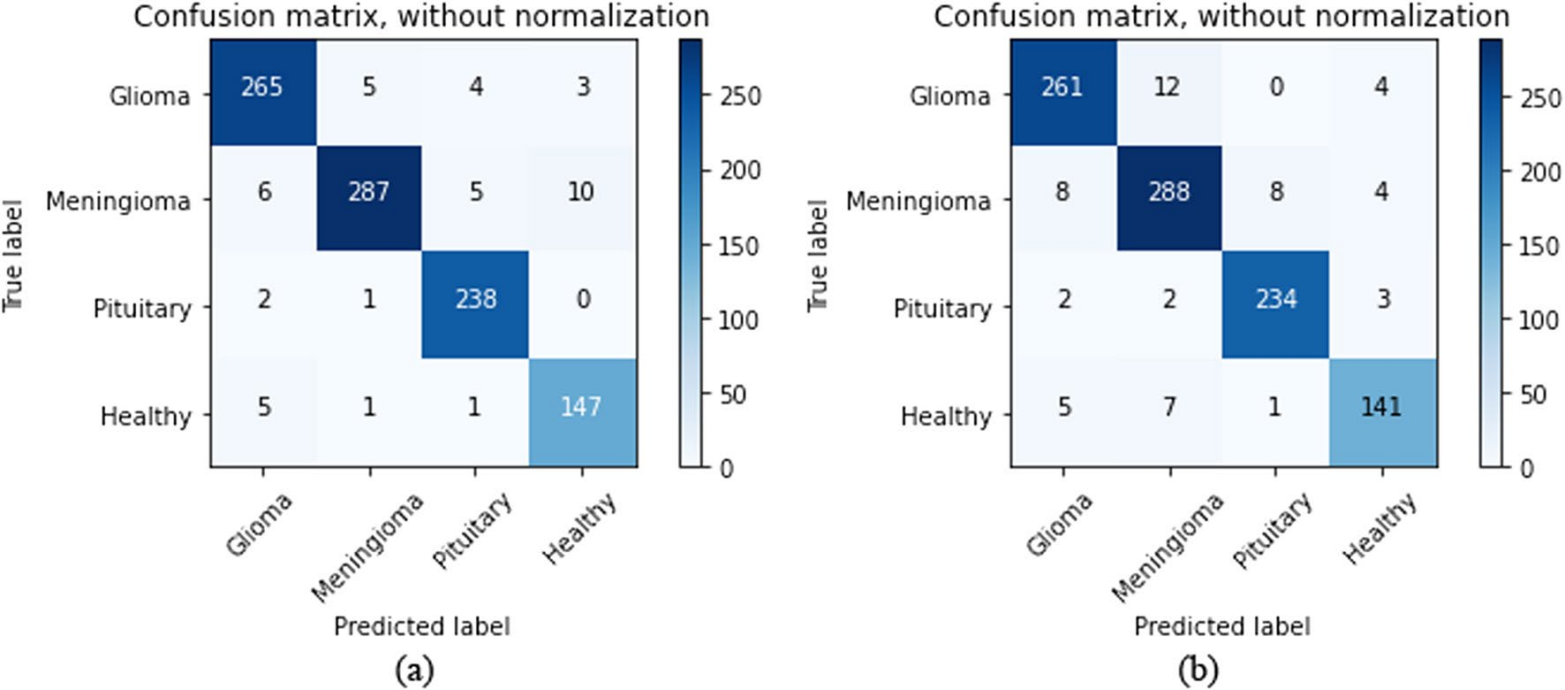
Confusion matrix analyses of the proposed model representing TP, TN, FP, and FN ratio obtained from the testing dataset of the **a** 2D CNN, and **b** convolutional auto-encoder neural network

In the field of artificial intelligence, a confusion matrix is a matrix in which the performance of relevant algorithms is visualized. Each matrix column represents the predicted value of instances, and each row represents the actual (true) value of instances. This matrix justifies its appellation that allows us to see whether there are confusing results or overlaps between the classes. In medical research, it is significantly important to reduce the false positive and false negative outcomes in the modeling process. The impact of false positive and false negative rates is shown in Fig. 7.

**Fig. 8:**
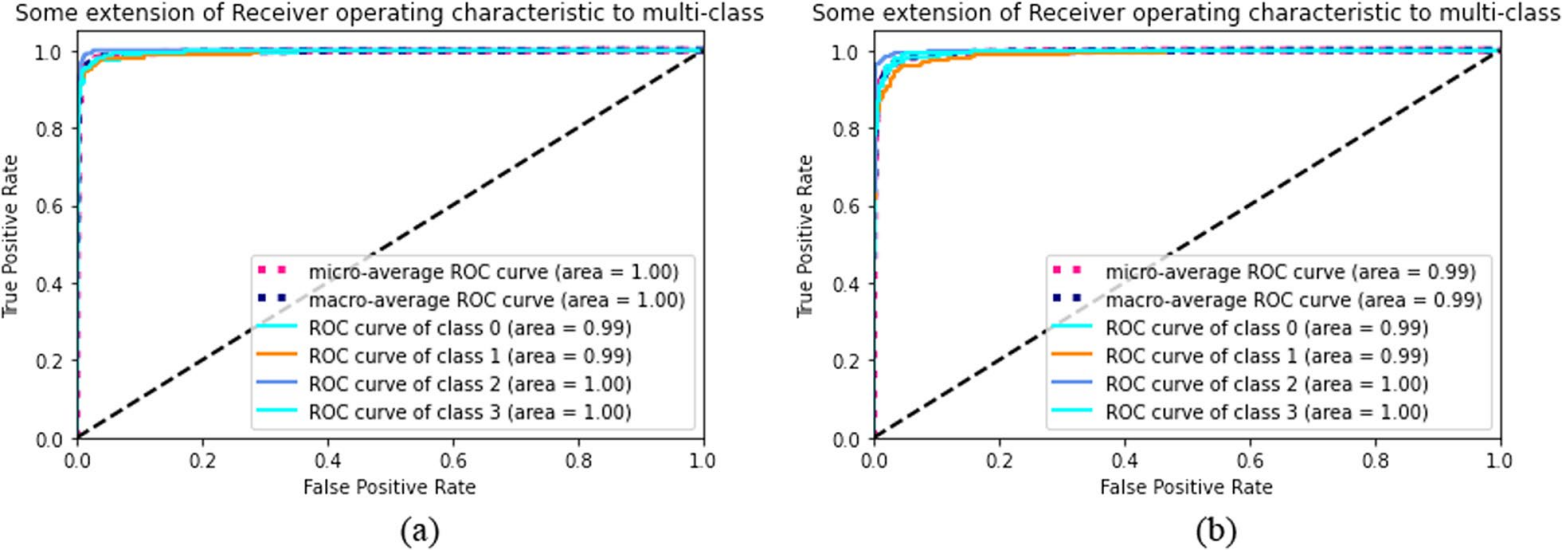
Roc plots of the **a** 2D CNN, and **b** convolutional auto-encoder neural network

Figure 8 presents the ROC curves of the proposed models along with classes 0, 1, 2, and 4 of the brain tumor classification models. The ideal point is observable for both class 0 and class 1.

**Fig. 9:**
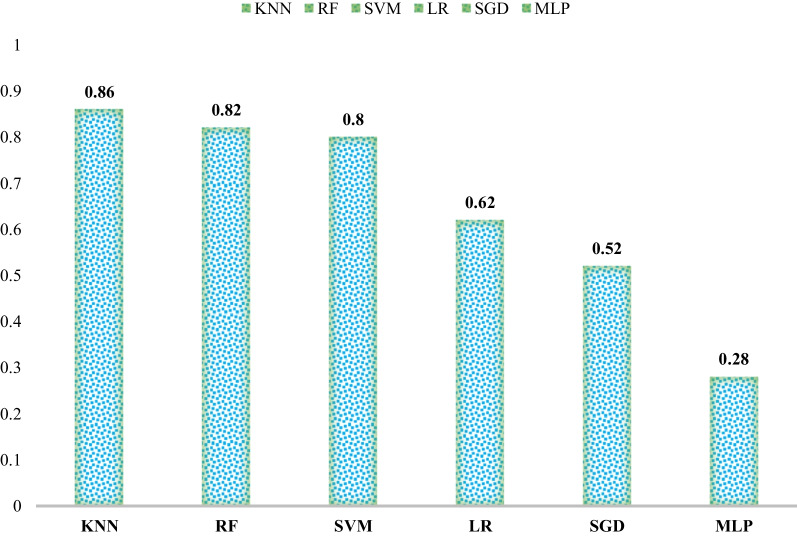
Comparison of classification accuracy rates of machine learning classifiers

The outcomes of classical machine learning classifiers like Support Vector Machine (SVM), Logical Regression (LR), Random Forest (RF), Nearest Neighbor (NN), Stochastic Gradient Descent (SGD), and Multilayer Perceptron (MLP) were compared and classified into four classes. The obtained accuracy rates were 86% for NN, 82% for RF, 80% for SVM, 62% for LR, 52% for SGD, and 28% for MLP. Figure 9 shows the comparison of these results. The precision, recall, and F-measure for each set of glioma, meningioma, pituitary gland tumor, and healthy brain images were calculated by these methods and are summarized in Table 8. For glioma tumor images, the highest precision was obtained by MLP (100%), the highest recall by KNN (90%), and the highest F-measure by KNN (87%). For meningioma tumors, the highest precision was obtained by KNN (93%), the highest recall by MLP (81%), and the highest F-measure by KNN (86%). For pituitary gland tumors, the highest precision was obtained by KNN (91%), the highest recall by RF (95%), and the highest F-measure by KNN (91%). For healthy brains, the highest precision was obtained by RF and SVM (83%), the highest recall by KNN (88%), and the highest F-measure by KNN (82%).

**Table 8:** Precision, recall, and F-measure of machine learning classifiers for the four classes of glioma, meningioma, pituitary gland tumors, and healthy brain

	KNN	RF	SVM	LR	SGD	MLP
*Glioma*						
Precision	0.84	0.91	0.78	0.61	0.63	**1.00**
Recall	**0.90**	0.75	0.79	0.61	0.52	0.01
F-measure	**0.87**	0.83	0.79	0.61	0.57	0.02
*Meningioma*						
Precision	**0.93**	0.80	0.79	0.61	0.65	0.32
Recall	0.80	0.80	0.73	0.60	0.23	**0.81**
F-measure	**0.86**	0.80	0.76	0.60	0.34	0.46
*Pituitary gland tumor*						
Precision	**0.91**	0.76	0.83	0.74	0.48	0.00
Recall	0.92	**0.95**	0.93	0.76	0.90	0.00
F-measure	**0.91**	0.84	0.88	0.75	0.63	0.00
*Healthy brain*						
Precision	0.77	**0.83**	**0.83**	0.52	0.43	0.14
Recall	**0.88**	0.78	0.75	0.52	0.55	0.19
F-measure	**0.82**	0.81	0.79	0.52	0.48	0.16

We bolded the parameters that had the best performance for each group

The results of one-way ANOVA for the three parameters of precision, recall, and F-measure in eight modeling methods showed a statistically significant difference between the methods (*p*-value < 0.001) (Table 9). LSD post hoc test results showed a significant difference between the means of precision, recall, and F-measure in the two methods presented in this study (2D CNN and convolutional auto-encoder) and the means of the three methods LR, SGD, and MLP (*p*-value < 0.05). The mean F-measure parameter of the 2D CNN method, in addition to the three methods mentioned, was also significantly different from SVM (*p*-value < 0.05) (Table 10).

**Table 9:** Results of one-way analysis of variance (ANOVA) comparing precision, recall, and F-measure

	ANOVA				
	Sum of squares	*df*	Mean square	*F*	Sig
*Precision*					
Between groups	1.212	7	.173	6.090	.000
Within groups	.682	24	.028		
Total	1.894	31			
*Recall*					
Between groups	1.611	7	.230	7.343	.000
Within groups	.752	24	.031		
Total	2.364	31			
*F-measure*					
Between groups	2.033	7	.290	30.910	.000
Within groups	.226	24	.009		
Total	2.258	31

**Table 10:** Multiple comparisons between methods

Dependent variable	(I) method	(J) method	Mean difference (I–J)	SE	Sig	95% Confidence interval	
						Lower bound	Upper bound
Precision	2D CNN	LR	.32750*	.11921	.011	.0815	.5735
		SGD	.40000*	.11921	.003	.1540	.6460
		MLP	.58250*	.11921	.000	.3365	.8285
	Convolutional auto-encoder	LR	.32250*	.11921	.012	.0765	.5685
		SGD	.39500*	.11921	.003	.1490	.6410
		MLP	.57750*	.11921	.000	.3315	.8235
Recall	2D CNN	LR	.33500*	.12520	.013	.0766	.5934
		SGD	.40750*	.12520	.003	.1491	.6659
		MLP	.70500*	.12520	.000	.4466	.9634
	Convolutional auto-encoder	LR	.32000*	.12520	.017	.0616	.5784
		SGD	.39250*	.12520	.004	.1341	.6509
		MLP	.69000*	.12520	.000	.4316	.9484
F-measure	2D CNN	SVM	.14500*	.06854	.045	.0035	.2865
		LR	.33000*	.06854	.000	.1885	.4715
		SGD	.44500*	.06854	.000	.3035	.5865
		MLP	.79000*	.06854	.000	.6485	.9315
	Convolutional auto-encoder	LR	.32000*	.06854	.000	.1785	.4615
		SGD	.43500*	.06854	.000	.2935	.5765
		MLP	.78000*	.06854	.000	.6385	.9215

* The mean difference is significant at the 0.05 level

## Discussion

The main objective of the current study was to develop two various deep learning networks and six machine learning techniques to classify MRI images into three classes of brain tumors (glioma, meningioma and pituitary gland tumor) and one class of healthy brain. The applied image dataset was publicly available at GitHub with 3264 T1-weighted contrast-enhanced magnetic resonance imaging (MRI) images.

According to the literature, some studies have used the famous T1-weighted contrast-enhanced MRI dataset (Figshare dataset), which contained 3064 MRI images of the human brain for tumor detection with computational approaches like neural networks. Studies using this dataset for the classification of brain tumors are listed in Table 11. It should be noted that the study employed another dataset that included 3264 MRI images. This dataset contained four categories of MRI images, namely glioma, meningioma, and pituitary gland tumors and healthy brains (no tumors). Badža and Barjaktarović [5] conducted brain tumor detection using a CNN developed in MATLAB R2018a. Their proposed CNN had two convolutional layers of 64- and 16-filter lengths. The classification block had two fully connected layers: the first representing the flattened output of the max-pooling layer and the second having an equal number of hidden units to the number of tumor classes. The best result was reported as 95.40% for record-wise cross-validation for augmented images. Nonetheless, the highest accuracy obtained from the mentioned study (95.40%) with a value of 1.07 is less than our proposed 2D CNN. The execution time of our study was longer because of the complexity and high frequency of layers in the network, which justified the good accuracy we obtained. The longer execution time in the current study can be explained by the number of hidden layers, the pooling layers, and the batch sizes. It should be noted that the training of deeper networks requires extra time than the training of shallower or simpler networks [35].

**Table 11:** Comparative analysis of proposed work with previous works

Contribution	Features employed	Type of classifier (s)	Dataset	Technical environment	Accuracy (%)	k-Fold cross-validation method/data division
Badža et al. [5]	Elementary features- model based	CNN (with two convolution layers)	T1-weighted contrast-enhanced MRI (Figshare dataset)	MATLAB	96.56	60% data in training, 20% in validation, 20% in test and tenfold cross-validation
Pashaei et al. [27]	Elementary features- model based	CNN (with four convolution layers)	T1-weighted contrast enhanced MRI (Figshare dataset)	–	93.68	70% data in training, 30% in testing and tenfold cross-validation
Gumaei et al. [23]	GIST features	FNN (feedforward neural network)	T1-weighted contrast enhanced MRI (Figshare dataset)	MATLAB	94.23	70% data in training, 30% in testing and fivefold cross validation
Afshar et al. [21]	Elementary features- model based	CapsNet	T1-weighted contrast enhanced MRI (Figshare dataset)	Keras package, with Tensorflow	86.56	–
Rehman et al. [22]	Fine-tune/Freeze-AlexNet, GoogLeNet, and VGG16	SVM	T1-weighted contrast enhanced MRI (Figshare dataset)	MATLAB	98.69	70% of data in training, 15% for validation, and 15% in testing
Abiwinanda et al. [28]	Elementary features- model based	CNN (with two convolution layers)	T1-weighted contrast enhanced MRI (Figshare dataset)	Keras package, with Tensorflow	84.19	–
Proposed approaches	Elementary features-model based	Two CNNs	Brain tumor classification (MRI): four classes	Keras package, with Tensorflow	96.47	90% data in training, 10% in testing
					95.63	

In another research, a CNN and an extreme learning machine were applied to diagnose brain tumors. The proposed model utilized four convolution layers and batch-normalization layers with 16-, 32-, 64- and 128-filter lengths (3*3). Four ReLU layers and three max-pooling layers were used in the proposed CNN with stride size [2, 2]. The model only had one fully connected layer with three types of classes. Feature vectors extracted by the mentioned convolution and layers were used as the input of KE-CNN (kernel CNN). The KE-CNN had 91.28% accuracy for classifying brain tumors [27]. However, our proposed 2D CNN and auto-encoder network achieved 96.47% and 95.63% accuracy, respectively. We used several layers of convolution for both networks and created complex networks, which can be justified by the large volume of data we used to increase classification accuracy. In comparison, other studies used networks with a small number of layers or a small amount of data [36].

In general, by comparing the two networks used in the current study, it can be concluded that the 2D CNN operated with 1% more accuracy than the auto-encoder network. Although the 2D CNN is more straightforward than the auto-encoder network, it performed better in feature extraction and learning, and according to what was previously mentioned, it uses all the parameters for learning [37]. The execution time (the duration of each epoch) or the runtime of the proposed convolutional network is less than that of the auto-encoder network. Therefore, the use of ordinary hardware and memory can be enough to run our proposed 2D CNN. One of the most notable differences between the current study and others is the use of six machine learning bribes to classify brain tumor images. SVM, NN, RF, SGD, LR, and MLP were developed for diagnosing brain tumors accurately.

In [23], researchers used a hybrid feature extraction approach with regularized extreme learning machine to classify types of brain tumors. Their method works by extracting the main features of brain images, and then applying principal component analysis to compute a covariance matrix of features. In the last step, a RELM is developed for diagnosing brain tumors into three classes (meningioma, pituitary, glioma). This method achieved 94.23% accuracy, which is not optimal compared to the results obtained from the networks designed in the current study.

A capsule network was employed in another study to classify brain tumors. Due to its good performance, the segmented tumor regions were applied as the inputs of the proposed capsule net. This method was implemented on Python 2.7, based on the Keras Library, using the Adam optimizer. Capsule net reached 86.56% accuracy for classifying segmented tumor regions and 78% accuracy for whole-brain tissue as input [21]. The researchers varied the feature maps in the convolutional layer of CapsNet in order to enhance accuracy; however, they achieved the highest accuracy of 86.56% using 64 feature maps with one convolutional layer of CapsNet. The network that employs only the tumor region or some other segmented part as input performs better in terms of execution speed. It also demands segmentation methods or a dedicated specialist to sign those parts [3, 29, 30]. The most favorable outcome in the research utilizing the segmented image parts as inputs has been presented by Tripathi and Bag [38], with 94.64% accuracy. They used features as inputs of classifiers extracted from the segmented brain tissue in the image. They checked their proposed method employing a fivefold cross-validation technique.

Like the approaches proposed in the current study, Rehman et al. performed the preprocessing of images with contrast improvement and dataset augmentation to reduce the occurrence of over-fitting and increase the database samples. Three types of CNNs (AlexNet, VGGNet, and GoogleNet) with an SVM model were utilized to diagnose brain tumors. The fine-tuned VGG16 network obtained the highest accuracy of 98.69% for the classification target [22]. In comparison, our developed methods 2% and 3% less accurate, respectively, than VGG16. This network is an intense and very deep network with 138 million weights, requiring complex hardware for calculating real-time performance [39]. Notably in this study, similar to ours, the researchers utilized various data augmentation methods to increase the size of the training dataset, such as rotating and flipping with raw MRI images. Data augmentation aims to enhance network performance by intentionally creating more training data from the original data.

In another study [28], CNNs were molded to determine the three most common types of brain tumors (i.e., glioma, meningioma, and pituitary gland tumors). In this study, researchers applied five different architectures of CNN for the classification of brain tumors and reported the highest accuracy for architecture 2. This architecture's training and validation accuracies were 98.51% and 84.19%, respectively. Architecture 2 is comprised of two convolutional layers, ReLU layer, and max-pooling with 64 hidden neurons. The testing accuracies of the developed networks in the current study were more significant than the accuracy of this architecture; nevertheless, this architecture has the capacity to overfit using a small learning rate or lower amount of training and testing data.

The current work is a pioneer study to develop two deep CNNs with optimal learning parameters and high accuracy. We also compared six machine learning techniques applied to classify brain tumors and healthy brains (no tumor). To better compare the previous studies conducted in this area, some key results are given in Table 11.

A limitation of medical image classification is the small size of medical image databases. This limitation, in turn, restricts the availability of medical images for training deep neural networks. One way to deal with this challenge in our study is to apply data augmentation techniques to create new brain tumor lesions through scaling and rotation, which may cause class imbalance. In addition, in this study, our primary plan was to train the networks using local images of a hospital, but the problem of labeling the images prevented this from being implemented. Labeling cancer images is not only time-consuming but also requires a high level of expertise that is challenging in brain tumor analysis. In future works, considering the importance of rapid and accurate diagnosis of brain tumors without latency, we will investigate the constructions of other robust deep neural networks for brain tumor classification with less execution time and more simplicity. Hence, full machine learning and deep learning algorithms can be implemented as future enhancements. Furthermore, the proposed techniques can be used to detect different forms of cancers in MRI or Computed Tomography (CT) scan.

## Conclusion

One of the areas of use for artificial intelligence and machine learning is the health domain. Deep networks are currently being designed and developed to detect diseases based on imaging. In order to do this, we have proposed computational-oriented methods to classify brain tumors. In our study, a novel 2D CNN architecture, a convolutional auto-encoder network, and six common machine-learning techniques were developed for brain tumor detection. This classification was conducted using a T1-weighted, contrast-enhanced MRI dataset, which includes three types of tumors and a healthy brain with no tumors.

According to the results and output shown in Figs. 6, 7 and 8, the proposed neural networks showed significant improvement over previous ones in detecting brain MRI image features and classifying them into three types of tumors and one class of healthy brain. The training accuracy of the proposed 2D CNN was found to be 96.47%, and the training accuracy of the proposed auto-encoder network was found to be 95.63%. In addition to the two-deep networks used in our study, six machine-learning techniques were also developed to classify brain tumors. The highest accuracies of 86%, 82% and 80% were attained for KNN, RF, and SVM, respectively. Comparing our networks with similar state-of-the-art methods shows that our proposed networks performed somewhat better with optimal execution time (maximum 15 min for 2D network and 25 min for auto-encoder network). The results of this study demonstrate that our proposed networks have an immeasurable generalization and high execution speed; therefore, they can be applied as effective decision-support agents for radiologists in medical diagnostics.

## Data Availability

All data generated or analyzed during this study are included in this published article. The link of the public MRI dataset that is used in this study is: https://www.kaggle.com/sartajbhuvaji/brain-tumor-classification-mri/.
